# Barriers, facilitators, and recommendations for childhood immunisation in Nigeria: perspectives from caregivers, community leaders, and healthcare workers

**DOI:** 10.11604/pamj.2022.43.97.35797

**Published:** 2022-10-25

**Authors:** Abisola Olaniyan, Chinwoke Isiguzo, Saheed Agbomeji, Olubunmi Akinlade-Omeni, Belinda Ifie, Mary Hawk

**Affiliations:** 1 Department of Behavioral and Community Health Sciences, Graduate School of Public Health, University of Pittsburgh, Pittsburgh, Pennsylvania, United States of America,; 2 Department of Primary Care, Derbyshire Community Health Service NHS Trust, Chesterfield, United Kingdom,; 3 Directorate of Medical Services and Disease Control, Lagos State Primary Healthcare Board, Lagos, Nigeria,; 4 Clinton Health Access Initiative, Lagos, Nigeria

**Keywords:** Childhood immunisation, vaccination, vaccine-preventable diseases

## Abstract

**Introduction:**

vaccination is one of the most successful and cost-effective public health interventions, significantly reducing childhood morbidity and mortality. In 2019, Nigeria had almost 2.5 million unvaccinated children. This study highlights barriers, facilitators, and recommendations for childhood immunisation uptake from various stakeholder perspectives.

**Methods:**

the study team conducted ten focus groups with mothers/caregivers and community leaders and nine semi-structured interviews with healthcare workers who provide routine immunisation services in Lagos State primary healthcare facilities. We performed a descriptive thematic analysis of the focus groups and semi-structured interviews.

**Results:**

study participants included 44 mothers/caregivers and 24 community leaders, and 19 primary healthcare workers in the State. Study participants reported barriers, facilitators, and recommendations for childhood immunisation uptake. Barriers include poor geographical and financial constraints to access healthcare services, inconducive health facility attributes, negative attitudes of health facility staff, vaccination misperceptions, and adverse events following immunisation. Facilitators include free immunisation service policy, optimal vaccine and device supply chain system, adequate knowledge of immunisation benefits and efficacy, vaccination outreaches, and provision of incentives to caregivers. Participants also made recommendations for implementation, including more awareness creation, use of community resources, employing more healthcare workers, frequent and optimal immunisation services and planning, and instituting a reminder system and defaulter tracking. **Conclusion:** our results can inform the development of interventions to improve childhood immunisation uptake. In addition, study findings can be employed to improve adult immunisation acceptance and uptake and other services provided within the primary healthcare setting.

## Introduction

Immunisation is one of the most cost-effective public health interventions, protecting the vaccinated and the unvaccinated via herd immunity [1,2]. Immunisation prevents approximately four to five million deaths annually [2], and an unprecedented wave of new vaccines in developing countries could dramatically reduce the global burden of disease. Vaccine-preventable deaths could further decrease by 1.5 million if optimal childhood immunisation coverage is attained [2]. WHO reports that 60% of the 23 million unvaccinated or partially immunised infants worldwide reside in just ten countries, including Nigeria [3]. Nigeria has introduced many efforts to increase vaccination coverage. However, only 23% of its children are fully vaccinated, far short of its 87% target, reflecting erratic progress of Nigeria's National Program on Immunisation (NPI) [4,5].

Lagos is the second most populous State, with 6.4% of the Nigerian population. It has 20 rural, semi-urban, or urban local government areas (LGAs) with residents of varying socioeconomic status. Lagos State has achieved full childhood immunisation coverage of 68.1% [4], far below the country's target, despite significant intervention by government and immunisation partners. Studies exploring determinants of Nigerian childhood immunisation coverage have primarily focused on maternal factors, demonstrating completeness of vaccination is significantly influenced by mothers' knowledge, ethnicity, and occupation [6-9]. Additional work is needed to understand barriers to childhood immunisation fully at both proximal and distal levels.

To our knowledge, no qualitative study to date describes barriers, facilitators, and recommendations for childhood immunisation in Nigeria from multi-stakeholders across all Local Government Areas, including rural, semi-urban and urban regions of Lagos State. Our study also utilised the socioecological model (SEM) as a framework informing our interview guides and qualitative analysis. We report the perceptions of caregivers, community leaders, and healthcare workers on immunisation-seeking behaviors of caregivers and identify facilitators to improve uptake. This work aims to elucidate opportunities to advance childhood immunisation rates in Nigeria.

## Methods

**Study design:** the study team utilised a qualitative descriptive approach to explore the barriers and facilitators of childhood immunisation uptake and recommend ways to increase uptake. We conducted focus groups and semi-structured interviews with caregivers (e.g., mothers and other adults caring for children), community leaders, and healthcare workers. Study design and methods are fully described elsewhere [10]. Briefly, we developed interview guides building on extant literature and the socioecological model (SEM).

**Setting and participants:** we used convenience sampling to recruit participants, aiming for statewide reach. Immunisation officials from the Lagos State Primary Health Care Board helped recruit healthcare workers and community leaders; these individuals recruited caregivers and more community leaders. Participants were approached face-to-face in the community and immunisation and postnatal clinics. Eligibility criteria were age ≥18 years, resident of Lagos State, and speaking English or Yoruba. Healthcare workers must have provided routine immunisation at health facilities. Researchers addressed ethical concerns by informing participants of the study purpose, potential risks and benefits of participation, their right to decline to participate or withdraw, incentives, and confidentiality of data. We collected verbal consent prior to data collection. The first author (Abisola Olaniyan), a female native Yoruba with qualitative research training and experience, conducted the interviews and focus groups in English or Yoruba using field notes to facilitate reflexivity. Interviews averaged 60 minutes and were audio-recorded and transcribed verbatim. Yoruba language transcripts were translated to English and reviewed for accuracy by the first author. Data collection continued until saturation was achieved.

**Data analysis:** we conducted an iterative process of thematic analysis employing an inductive approach via NVivo12. First, two team members read four transcripts and developed a set of codes grounded in our research questions. The study team discussed and refined codes until they were fully understood and agreed upon. Next, two team members separately coded two of the interviews, again refining codes and comparing coding structures until there was agreement. Finally, three team members coded the remaining transcripts, double-coding 8 of the 19 transcripts to ensure consistent code use. Emerging themes were discussed and reviewed along with field notes, then added to the codebook once agreement was reached. The team compiled and reviewed excerpts from coded transcripts, then discussed and analysed emerging and interrelated themes.

**Ethical considerations:** the Health Research and Ethics Committee of the Lagos State University Teaching Hospital approved the study protocol (Ref. No: LREC/06/10/1631). The protocol was deemed exempt from review by the University of Pittsburgh Institutional Review Board (IRB#: PRO18060037). We also received social approval from the Lagos State Primary Health Care Board (LSPHCB).

## Results

We completed ten focus groups with caregivers and community leaders (n=68, 6-8 participants per group) and nine interviews with healthcare workers (n=19, 1-3 people per interview). Participants described numerous barriers and facilitators to immunisation uptake and provided recommendations to increase coverage.

**Barriers:** lack of access to healthcare services was identified as a barrier, including infrastructure factors like bad roads and long distances to health facilities. Long distances increased costs of transportation, causing financial barriers to immunisation uptake: “*What also discourages people is the cost of transportation to the health center. My transport fare to the health center is N300; if I have not eaten today and don't have any more money, that would discourage me*” [female caregiver AK]. Participants discussed health facilities' characteristics that negatively impacted immunisation decisions, including physical attributes like insufficient seating or inadequate ventilation. “*...Some get discouraged by the way the health center looks. Some people go to the primary health center just because they can't go to waste money at private hospitals, and before you get to that health center with the child you are sitting down, you are not comfortable*” [female caregiver AK]. “*...Our major PHC is under construction, but where we are staying presently is not even good. When the rain is falling, you will see how all the mothers will just be running from one place to another. And if such a thing continues, I am sorry, we will not see them. We will lose most of our patients*” [female healthcare worker AL]. Participants also discussed inadequate drugs for patients, including drug stockouts, as a barrier since caregivers often receive vaccinations while accessing medications like paracetamol or multivitamins. “*There are plenty of children, but the drugs are not always enough; sometimes out of 100 children, there might be just 50 drugs, and then they will give others appointments for the next day*” [female community leader AK].

Insufficient healthcare staffing including strike actions, contributed to poor access and missed opportunities to vaccinate children. Staffing shortages also resulted in overcrowding and long waits, keeping some caregivers from seeking care due to time constraints from competing demands, including work. “*Our nurses are not enough so …We spend almost the whole day in the health center to immunise our children. ...Sometimes it is so crowded that you won't even get a place to sit*” [female caregiver PM]. “*....It is not easy. You get there (public health facility) very early, and sometimes you won't leave till late afternoon*” [female caregiver AF]. Healthcare workers' attitudes and skills deterred immunisation uptake for some. Participants discussed how healthcare workers' insults made them reluctant to return for subsequent immunisations. “*To be sincere with you, I don't really like the idea of going to the general hospital because the nurses are mostly rude to mothers that come in for delivery like people used to say. Although I did0n't give birth in a general hospital, I used to hear about it. I am saucy. So, I don't want to be insulted*” [female caregiver AL]. Participants said caregivers were insulted when they lost their wards' immunisation cards or when facilities were overcrowded and providers overworked. They also reported feeling disrespected by healthcare workers if they arrived late. “*All of them (healthcare workers and staff), they don't know how to talk. Imagine someone sitting, and the nurse tells the person that she lacks common sense or is stupid. So, these are what some mothers think that discourages them from taking their children there*” [female caregiver AK].

Another barrier to childhood immunisation uptake was misperceptions about immunisation. Due to widespread rumors and mistrust of Western medicine, some caregivers believe developed countries introduced immunisation into developing countries to make children sterile, cause fatal diseases, and reduce the population. Misinformation has been passed down through generations and among peers. “*What people say when we counsel them to go to the health center for immunisation, even with the door-to-door immunisation program. Some people still refused to take it. When we get to some people's doorstep, and they see us in green uniform, they lock their door firmly…when we started counseling them and asked their reasons for refusal. They said that it reduces their birth rate…That it causes infertility*” [female community leader LM]. Some participants discussed adverse immunisation events, including fever and injection site swelling, as causing concern for children's welfare, discouraging some from completing vaccination schedules. “*…There are some children who are always running temperature after they are brought for immunisation. Their head and body is always hot, and it makes them uncomfortable to the extent that the mother will be contemplating going back. It happened to me as well*” [female caregiver AL].

**Facilitators:** a major facilitator of childhood immunisation uptake is the provision of free immunisation services at government-owned health facilities. This was mentioned by many caregivers, community leaders, and healthcare workers as reducing the financial burden of immunisation. “*…The immunisation is free… It makes them come to access the immunisation, having it in mind that they are not going to pay any amount because some of them when they even come, they will tell you, 'I don't even have money to eat*” [female healthcare worker MU]. Some facilities provide free consumables like gloves and cotton wool, making them caregivers' preferred locations. However, while free services promote uptake, these can result in overcrowding in facilities, especially those with staff shortages, infrequent vaccination days, or inefficient scheduling.

Participants discussed government efforts to ensure availability of adequate vaccines and storage devices in health facilities as integral to vaccine distribution. The current vaccine and supply chain system (Push) in Lagos State has improved immunisation access. Vaccines are delivered via a stock management system, eliminating visiting vaccine storage facilities. One healthcare worker described this as follows: “*... they come to us, and we say it's not available..., it is discouraging, but now we have enough, and they have also made it easier by using this (push), so even if you cannot go to the facility, the NPI cold store, they come to us and supply us vaccines, so it's very convenient, and it is very easy to access*” [female healthcare worker SU]. Caregivers' knowledge of immunisation benefits and efficacy was reported as increasing uptake. Interviewer (I): “*So what other things encourage you to immunise your child?*” Participant (P): “*The diseases they prevent against Polio, measles, hepatitis, yellow fever..*.” [female caregiver AK]. I: “*What has made you decide to immunise your child now?*” P: “*Because I see the effectiveness of its work on children*” [female caregiver AK]. “*...If my child is immunised, when they are in class with other children, who are not immunised, the immunisation will prevent my children from contracting any disease from their classmates*” [female caregiver LM].

Caregivers discussed learning about immunisation benefits through community leaders, family members, peers, healthcare workers, and antenatal clinics. “*The nurses have educated us that immunisation is good for our children's health. Once we give birth, we need to immunise our baby. They tell us when to bring our children for immunisation. Before the naming ceremony of the baby, we take the baby for BCG injection against infection. When we get there, they lecture us on the benefit of immunisation, and they make us understand its essence. They let us know that it's good for the babies against germs and diseases without a side effect. So, after that one, they tell us to come with the baby after six weeks for another vaccine; because it is good for the baby. That is why people are trooping in*” [female caregiver IK]. Caregivers continue to learn about the vaccination schedule and immunisation during child welfare clinics. Songs are used to aid knowledge retention. Knowledge of vaccine benefits gave some caregivers a sense of relief: some described vaccination as an expression of love and the importance of immunising children as they are the future of the country. “*It's easy for me, oh! Because it will heal my baby and will prevent my baby from any disease, and it will give him strength and me too, I will be relieved when my baby is okay*” [female caregiver PM]. “*Immunisation is a way of preventing our childhood diseases, reducing the mortality rate, and with the assistance of immunisation now, at least we can achieve that. We are moving towards our goals for our children; they are the future of tomorrow*” [female caregiver AF]. Participants reported vaccination outreach improved access to services, especially in areas with geographic barriers. “*…Going house to house, door to door, calling them out and all, this makes it easy. If your child is sleeping, you can wake the child up, and he/she would be immunised because of the house-to-house program that the government created*” [female caregiver PM].

Healthcare workers and community leaders collaborated on outreach efforts: “*We do outreaches, where we pick a certain settlement like some people they don't come to the health center facility, so we pick a settlement that they have a large number of children in that settlement then we pick a particular day, but before we do that we send a community leader to go to those areas and announce to them the day we are coming, we have a fixed place where we do it, so before we go there in the morning the community leader will have gone there make advocacy, gather some of the women and their kids, then we give immunization*” [female healthcare worker SU]. Incentives, or “pluses” were seen as facilitators, especially for those with low socioeconomic status. Caregivers were given pampers, milk, and bleach during immunisation sessions and campaigns such as the Maternal, Newborn, and Child Health week (MNCH week) and National Immunisation Plus Days. Some facilities also coupled vaccine education with food demonstration classes where caregivers were taught how to prepare meals for their kids when weaning them. “*Won't you take your child for immunisation if you know you will get pampers or hypo (bleach detergent) or milk? Me, I would. It encourages me. During those programs, I always take my children*” [female caregiver AF]. Participants reported that in the past, mosquito nets were given as incentives, though net shortages now preclude this, and that congratulatory cards documenting completion of vaccine schedules conferred caregivers with a sense of pride. In addition, participants said these approaches might cost less and be more sustainable than diapers or other incentives.

**Recommendations:** to increase immunisation uptake, participants discussed further need to create awareness about vaccination benefits, the schedule, and access, including that it is free of charge, recommending the use of billboards, radio or television jingles, and community mobilisers at health facilities. “*What I want the government to continue is that they should not relent on the awareness through media like radio, television. They shouldn't stop because there are some mothers… I have seen someone staying around this area that said she didn't know where the health center was situated. I have also seen someone that stays on my street; she is pregnant. I asked her if she had registered at the health center. She said no that she goes to traditional home care. I told her, “In this present generation, you still go there.” It's alright; let's go to the health center. I brought her here*” [male community leader IK]. Participants also discussed the importance of early education during antenatal clinics and leveraging families and other caregivers to share the intergenerational information. P: “*So, I think the mobilisation drive should be encouraged*.” I: “*What do you mean by mobilisation drive?*” P: “*I mean to involve more parents to join… To encourage other mothers to join because if we remain silent, the fear is that these children will meet one way or the other like in a church, mosque*” [female caregiver LM].

They stressed the importance of collectivism, especially among caregivers, who could share information with peers. “*I still tell some people, 'Please, go out and immunise your children. I heard there's something going on. Make sure you take your kids for immunisation.” We still encourage mothers to immunise their wards despite the fact that we also go for it. We still remind them because it's so unfortunate that if we fail to encourage them, it can still bounce back on us. Because we can't guarantee that our children won't mix with them in school. They will interact with them because we can't rule that out. So! The best bet is for us to carry other people along. We often hear on the media like the radio and TV while the government sensitises the public. We also need to help by spreading the word*” [female caregiver LM]. For some, creating awareness was a way to create a culture of health, saying those sharing information should first understand objections and try to address these, thus taking a person-centered approach to developing relationships with caregivers to build trust and facilitate vaccine acceptance. “*Then our attitude too, our welcoming, our interpersonal relationship with them is very, very important. So, by the time we do that, we are like friends, they will trust us, and they will find it interesting coming to the health facility*” [female healthcare worker SU]. They also said information must be tailored to different stakeholders, linguistically specific, and culturally acceptable.

Study participants recommended the use of community resources for mobilisation, including traditional, religious, and market leaders, to assist with creating awareness. Participants perceived these community champions as better equipped to encourage caregivers to seek and accept immunisation services as they were older, more experienced and knowledgeable, and also well-known within communities. They were able to identify pregnant women within the community, talk about the importance of immunisation, and encourage vaccination. Additionally, their knowledge of community events helps with tracking children for immunisation follow-up and identifying hesitant caregivers. “*The community leaders are more in contact with the people. They know the people living in their area, and people will trust them more. The more people are interested in what you are saying, then they will encourage people that are against the government and immunisation. When they see the faces of mobilisers or community leaders that they know very well, the mothers will start asking when they are bringing the program*” [female caregiver AF]. Participants emphasised the importance of traditional and religious leaders in specific ethnic groups seen as more vaccine-hesitant due to intergenerational misperceptions; these leaders could provide culturally and linguistically acceptable information. *...They do employ people to work with mobilisers or jingle people so that they can be speaking in Hausa. So when they are where those Hausas are, they will speak in their language. And along that abattoir, when they want to work there, they normally go to meet their Dungi (traditional/religious leader). If you don't go to them, they will not allow it; they will tell you frankly that they are not goats, so you cannot immunise them. When they want to do any test, and if there's nobody to speak in Hausas to them, their Dungi or Seriki will say no*" [male community leader AK]. The importance of government compensation of community leaders was also emphasised.

Another recommendation was to employ more healthcare workers to not only reduce overcrowding and wait times but also minimise healthcare workers' workloads and stress. P: “*Our nurses are not enough, and we are pleading with the government to assist us by giving us more nurses…*” [female caregiver AF]. I: “*Do you think employing more nurses will encourage mothers to immunise their children?*” P: *“Yes. The crowd in the health center will not be as much. They will be able to answer mothers quickly, and we won't have to wait for too long. We spend almost the whole day in the health center to immunise our children. If there are more nurses, we will be able to get immunisation quickly and do other things that day. Also, we will be able to sit comfortably. Sometimes it is so crowded that you won't even get a place to sit*” [female caregiver AF]. Staffing increase should include employing more records personnel since record-keeping could delay immunisation sessions. “*We are short of staff because there have to be workers that will attend to the patients; workers that will collect the card and those that will take records. It could be two people. That's what is causing the delay... if the government can assist us, they should provide us with more staff*” [male community leader AL]. Lacking these personnel, nurses in some facilities both immunise children and document vaccinations, further slowing down processes. “*In a situation where we have hundreds of clients all over with maybe one nurse. You know it's not ideal. Because with this work, documentation is key. Any work done without documentation is not done. We have many registers that we will record in, and we don't have any records person, so we have to do it too... the workload is too much on someone because we need to do everything accurately...so, shortage of manpower is the problem; the government should help us employ records staff and more nurses*” [female healthcare worker AF].

Another recommendation was to increase the frequency of immunisation services and include weekends to improve access for caregivers with work constraints. They also proposed scheduling vaccination appointments based on healthcare worker availability to address overcrowding and wait times.

Finally, participants suggested the need for a reminder and defaulter-tracking system since some caregivers reported forgetting about appointments. This could be done with Short Message Service (SMS) reminders. Some caregivers said SMS reminders were previously helpful but had been discontinued despite their value. “*There was a time I used to get SMS reminders about when I am to immunise my child again. It helped me remember, but they stopped. I actually missed my next appointment because I was waiting for the reminder, and I didn't get it, and I had forgotten about his next immunisation date*” [female caregiver AK].

## Discussion

Our study highlights barriers, facilitators, and recommendations for childhood immunisation uptake from a multi-stakeholder perspective. Our results show the interplay of the multi-levels of the socioecological model by highlighting individual, social network, organisational (health facility and health worker), community and policy-level factors that drive immunisation uptake and potential interventions to enhance coverage. Our findings substantiate those of other studies describing immunisation barriers related to geographical challenges, health facility conditions, vaccination misperceptions, and Adverse Events Following Immunisation [7,11-13]. Like other studies, our participants emphasised the importance of community champions, educating caregivers, SMS reminders and adequate staff numbers to improve immunisation uptake [12,14-16].

Our study also provides recommendations to improve vaccine uptake at the health system-level, uch as implementing a defaulter tracking system and conducting frequent and conveniently timed immunisation services to improve childhood immunisation uptake. We also highlight the significance of culturally- and linguistically appropriate interventions for Nigeria's diverse ethnic groups and cultures, which can be achieved through the involvement of community members. Finally, our study findings substantiate the need for multi-pronged interventions addressing various levels of the socioecological model to enhance childhood immunisation uptake.

Our study is not without limitations, including the use of convenience sampling. We recruited all participants from Lagos State, so responses might vary in States with different policies and programs. Nonetheless, this study is the first to explore barriers, facilitators and recommendations for childhood immunisation uptake in Lagos State from a multi-stakeholder perspective.

## Conclusion

Implementing participants' recommendations, enhancing noted facilitators, and addressing reported barriers may improve childhood immunisation coverage in Nigeria and support the uptake of new vaccines introduced in the country. Overall, the application of our findings may help the country attain its immunisation target and ultimately reduce childhood morbidity and mortality from vaccine-preventable diseases.

**Funding:** this study received funding support from the Center for Global Health, University of Pittsburgh Graduate School of Public Health, and the Global Health Student Association, University of Pittsburgh Graduate School of Public Health. The funders had no role in the study's design, data collection, data analyses, interpretation of data, or manuscript preparation.

### What is known about this topic

- Childhood immunisation is an essential public health program that reduces childhood morbidity and mortality due to vaccine-preventable diseases;
- Childhood immunisation coverage is low in Nigeria.

### What this study adds

- This study provides information about the barriers to childhood immunisation from the perspective of all stakeholders involved in childhood immunisation uptake decision-making; barriers reported include poor access to healthcare services due to bad roads or long distances, inadequate healthcare staff and medications, poor interpersonal communication skills of healthcare workers, misperceptions about immunisation, and the adverse events following immunization;
- Study findings also identified facilitators to childhood immunisation, such as free immunisation services, availability of adequate vaccines in healthcare facilities, and the caregiver's knowledge about the importance of vaccination; other facilitators reported include the conduct of immunisation outreaches, especially in hard-to-reach communities and the provision of incentives to caregivers during immunisation sessions;
- Our study participants also provided recommendations to improve childhood immunisation uptake; these recommendations include utilising community leaders for awareness creation and mobilisation, employing more healthcare workers, and institutionalising a reminder and defaulter tracking system.
